# Left Ventricular Unloading During Veno-Arterial Extracorporeal Membrane Oxygenation (VA-ECMO) in Toxic Shock: Successful ECpella Support After Massive Amlodipine Ingestion

**DOI:** 10.7759/cureus.108778

**Published:** 2026-05-13

**Authors:** Manoj Sharma, Mays Tawayha, Ngoc Thai Kieu, Husam Katib, Wassim Mosleh

**Affiliations:** 1 Clinical Skills, St. George's University School of Medicine, St. George's, GRD; 2 Internal Medicine, University at Buffalo, Buffalo, USA; 3 Internal Medicine, Sisters of Charity Hospital, Buffalo, USA; 4 Geriatric Medicine, HCA Florida Aventura Hospital/Nova Southeastern University, Aventura, USA; 5 Cardiology, Dartmouth-Hitchcock Medical Center, Lebanon, USA

**Keywords:** calcium channel blocker overdose, impella, mechanical circulatory support, toxic shock, va-ecmo

## Abstract

Massive overdose of calcium channel blockers and angiotensin-converting enzyme inhibitors can result in refractory mixed vasoplegic and cardiogenic shock. We present a case of a 65-year-old woman with severe amlodipine and lisinopril overdose requiring veno-arterial extracorporeal membrane oxygenation (VA-ECMO). Despite improved systemic perfusion, the patient developed left ventricular distension characterized by reduced pulsatility, elevated pulmonary capillary wedge pressure, and severely depressed left ventricular function. Early placement of an Impella device for active left ventricular unloading resulted in improved hemodynamics, restoration of pulsatility, and recovery of ventricular function. The patient was successfully weaned from mechanical support with full clinical recovery. This case highlights the importance of hemodynamic-guided left ventricular unloading during VA-ECMO in toxicologic shock and supports the role of ECpella in selected patients.

## Introduction

An overdose of cardiovascular drugs such as dihydropyridine calcium channel blockers (for example, amlodipine) and angiotensin-converting enzyme (ACE) inhibitors (such as lisinopril) can cause life-threatening hemodynamic collapse [1]. This condition is characterized by severe vasodilation, myocardial depression, and arrhythmias.

Initial management of these overdoses typically includes high-dose insulin euglycemia therapy (HIET), vasopressors, and temporary pacing when needed [1]. In severe toxicity, these therapies may be insufficient, and veno-arterial extracorporeal membrane oxygenation (VA-ECMO) may be required for mechanical circulatory support [2,3]. VA-ECMO is often used as a last resort for shock caused by drug overdose; however, it may not be sufficient on its own. It can lead to complications such as left ventricular (LV) distention, reduced ejection fraction, pulmonary edema, pericardial effusion, and impaired coronary perfusion. To mitigate these effects, VA-ECMO may be combined with an LV assist device such as the Impella. This combined approach, termed "ECpella," is commonly used in non-toxic cardiogenic shock but has rarely been reported in cases of poisoning [2,3].

This report describes a case of severe amlodipine and lisinopril overdose that resulted in mixed vasoplegic and cardiogenic shock requiring VA-ECMO. Early recognition of LV distension physiology prompted the use of an Impella device, contributing to the limited evidence supporting ECpella use in toxicologic shock.

## Case presentation

A 65-year-old woman presented to the emergency department after intentionally ingesting a large quantity of her antihypertensive medications. She reported taking approximately 20-30 tablets of amlodipine (10 mg each) and 20-30 tablets of lisinopril (20 mg each). While in the emergency department, she experienced a brief episode of seizure-like activity without loss of consciousness or a postictal state. Initially, she appeared hemodynamically stable; however, she subsequently developed bradycardia and worsening mental status, ultimately requiring intubation for airway protection.

The patient received aggressive medical therapy including intravenous calcium, initiation of a high-dose insulin euglycemia protocol, and escalating doses of norepinephrine, epinephrine, vasopressin, and angiotensin II. A bolus of methylene blue was also administered due to persistent hypotension. Despite these interventions, the patient remained in refractory shock. Cardiothoracic surgery was consulted emergently, and the patient was transferred to the cardiovascular intensive care unit (CVICU), where veno-arterial ECMO was initiated for circulatory support.

VA-ECMO was initiated at approximately 3.5 L/min, resulting in improved systemic perfusion. However, arterial line tracings demonstrated markedly limited pulsatility, with a pulse pressure of 9-11 mmHg. A pulmonary artery catheter was subsequently placed, revealing an elevated pulmonary capillary wedge pressure (PCWP) of 18-19 mmHg, consistent with increased LV filling pressures and impaired ventricular ejection. Right atrial pressure and pulmonary artery pressures were measured via the pulmonary artery catheter to guide hemodynamic management. Transthoracic echocardiography confirmed severely reduced LV systolic function with an estimated ejection fraction of 15%-20%, multiple akinetic segments, and minimal aortic valve opening.

Given concerns that continued ECMO support could worsen pulmonary congestion and intracardiac stasis and potentially impair myocardial recovery, placement of an Impella device for active LV unloading was pursued. Following Impella placement, invasive hemodynamic monitoring demonstrated improved systemic perfusion. Cardiac index ranged between 3.3 and 3.8 L/min/m² with systemic vascular resistance (SVR) values between 749 and 842 dyn·s/cm⁵. Central venous pressure ranged from 8 to 14 mmHg, and pulmonary artery pressures were 32/14 mmHg. Mixed venous oxygen saturation ranged from 86% to 90%. ECMO flow was maintained at approximately 3.5 L/min while the Impella CP provided an additional 1.6-2.1 L/min of forward flow at a P2 support level. These findings suggested adequate systemic perfusion with improved ventricular unloading following Impella placement.

Following Impella placement, LV filling pressures improved, arterial pulsatility increased, and aortic valve excursion was restored. Vasopressor requirements also decreased, and lactate levels progressively declined, indicating improved end-organ perfusion. Hemodynamic parameters recorded during combined VA-ECMO and Impella support are summarized in Table 1.

**Table 1:** Hemodynamic parameters during combined VA-ECMO and Impella (ECpella) support. VA-ECMO: veno-arterial extracorporeal membrane oxygenation; PCWP: pulmonary capillary wedge pressure; ECMO: extracorporeal membrane oxygenation

Parameter	Value
ECMO flow	3.5 L/min
Impella flow	1.6-2.1 L/min
Pulse pressure (pre-Impella)	9-11 mmHg
PCWP	18-19 mmHg
Cardiac index	3.3-3.8 L/min/m²
Systemic vascular resistance	749-842 dyn·s/cm⁵
Central venous pressure	8-14 mmHg
Pulmonary artery pressure	32/14 mmHg
Mixed venous oxygen saturation	86%-90%

Over the subsequent six days, serial echocardiograms demonstrated gradual improvement in LV function. Ejection fraction improved from 15%-20% to 41%-45% while the Impella device remained in place and ultimately normalized to 55%-65% prior to device removal. After recovery of myocardial function, the patient was successfully weaned from both VA-ECMO and Impella support. She was subsequently extubated with intact neurologic function and continued to recover without recurrent hemodynamic instability.

## Discussion

Impella is typically used in cardiogenic shock characterized by elevated SVR, where active LV unloading improves forward flow. In contrast, ACE inhibitor toxicity can cause vasodilatory shock, raising concern that Impella placement could worsen hypotension by reducing afterload. However, in the setting of VA-ECMO, retrograde aortic flow increases effective afterload and may precipitate LV distension in patients with impaired myocardial contractility. In our patient, shortly after ECMO initiation, the arterial pulse pressure narrowed to 9-11 mmHg, and PCWP increased to 18-19 mmHg, indicating impaired ventricular ejection and elevated filling pressures. Hemodynamic monitoring after Impella placement demonstrated cardiac index values of 3.3-3.8 L/min/m² with SVR ranging from 749 to 842 dyn·s/cm⁵, suggesting that vasoplegia was not the dominant physiology at that stage. These findings supported the decision to proceed with active LV unloading using Impella despite concurrent ACE inhibitor toxicity.

A massive dose of a dihydropyridine calcium channel blocker can cause profound vasoplegia and myocardial depression, while concurrent ACE inhibitor ingestion limits compensatory increases in SVR [1]. Consequently, patients may develop a shock state that is extremely difficult to manage with conventional therapies such as high-dose insulin, vasopressors, calcium, and methylene blue. Although multiple reports describe ECMO use in severe calcium channel blocker overdose [3-5], few describe a strategy for mechanical LV unloading with devices such as Impella. As a result, the toxicology literature contains limited information regarding the potential benefits and risks of this approach.

An important limitation of VA-ECMO is the retrograde aortic flow it generates, which increases LV afterload [2] and can worsen LV distension in patients with severely impaired contractile function. LV distension is associated with elevated filling pressures, pulmonary edema, impaired coronary perfusion, and intracavitary stasis. These complications have been described in both toxicologic and non-toxic cardiogenic shock. In most cases, ECMO alone restores circulatory stability without clinically significant LV distension, and unloading is therefore not routinely required. Additionally, the combined use of Impella and VA-ECMO is technically more complex than ECMO alone.

In our patient, mixed vasoplegic and cardiogenic shock was present with profound myocardial depression, rising PCWP, loss of arterial pulsatility, and minimal native ventricular ejection early after ECMO initiation. Pulse pressure narrowed to 9-11 mmHg within one hour of VA-ECMO support, and pulmonary artery catheterization demonstrated a PCWP of 18-19 mmHg, consistent with rising LV end-diastolic pressure. Echocardiography confirmed severely depressed LV function with an LV ejection fraction (LVEF) of 15%-20% and minimal aortic valve opening. These findings prompted placement of an Impella CP device to actively decompress the LV, reduce LV filling pressures, restore pulsatility, and improve coronary perfusion, ultimately facilitating ECMO weaning.

Early LV unloading during ECMO (“ECpella”) has been increasingly reported in non-toxic cardiogenic shock and has been associated with improved outcomes, reduced pulmonary congestion, and lower mortality. However, published experience with combined ECMO and Impella support in toxicologic shock remains limited. Recent reports of ECMO use in overdose, including cases of dihydropyridine toxicity and a broader series of cardiotoxic poisonings, provide a physiological rationale for escalation to mechanical circulatory support in reversible toxicologic conditions. Our case extends this evidence by demonstrating that objective hemodynamic indicators such as pulse pressure and PCWP can guide timely LV unloading and facilitate full myocardial recovery.

Several limitations should be acknowledged. As a single-patient report, the observations described here cannot be generalized to all cases of toxin-induced refractory shock, and the temporal association between Impella placement and clinical improvement does not establish causation. The published experience with combined VA-ECMO and Impella support in toxicologic shock remains small, and direct comparison with prior cases is therefore constrained by heterogeneity in toxin class, ingestion burden, timing of mechanical support, and reporting of hemodynamic data. Long-term follow-up beyond the index hospitalization, including outpatient assessment of LV function and functional status, was not available for this patient and represents an important gap. Finally, although no major device-related complications were encountered during this admission, the combined use of VA-ECMO and Impella carries recognized risks, including vascular injury, bleeding, hemolysis, limb ischemia, and device malposition, that should be weighed against the potential hemodynamic benefits when this strategy is considered.

## Conclusions

In this patient with severe mixed vasoplegic and cardiogenic shock due to massive amlodipine and lisinopril overdose, early VA-ECMO combined with active LV unloading using Impella resulted in full myocardial recovery and a favorable clinical outcome. This case supports consideration of ECpella in toxin-induced refractory shock and highlights the importance of early recognition of LV distension. Importantly, decisions regarding LV unloading during VA-ECMO should be guided by real-time hemodynamic parameters such as pulse pressure and PCWP rather than toxin class alone.
